# Unexpected bioprosthetic mitral valve thrombus during left ventricular assist device implantation

**DOI:** 10.1186/s40981-017-0086-5

**Published:** 2017-04-17

**Authors:** Tatsuyuki Imada, Sho Carl Shibata, Kenta Okitsu, Yuji Fujino

**Affiliations:** Department of Anesthesiology and Intensive Care Medicine, Osaka University Graduate School of Medicine, 2-2, Yamadaoka, Suita, Osaka, 565-0871 Japan

**Keywords:** Bioprosthetic valve thrombosis, Mitral stenosis, Mitral valve replacement

## Abstract

Acute bioprosthetic valve thrombosis can occur after surgery and sometimes cause hemodynamic instability and cardiogenic shock. Risk factors for bioprosthetic valve thrombosis are hypercoagulability, atrial fibrillation, atrial dilatation, low cardiac function, and lack of anticoagulation therapy. The authors present a case of severe mitral stenosis due to bioprosthetic valve thrombus. The patient was diagnosed with dilated-phase hypertrophic cardiomyopathy and underwent mitral valve replacement. He required venoarterial extracorporeal membrane oxygenation (VA-ECMO) due to extremely low cardiac output and was scheduled for left ventricular assist device (LVAD) implantation. Transesophageal echocardiographic examination before LVAD implantation revealed severe mitral stenosis due to bioprosthetic mitral valve thrombus, which was not detected by transthoracic echocardiography in the intensive care unit and contributed to the low cardiac function. The thrombus was removed through an unscheduled left atriotomy before LVAD implantation. The possibility of bioprosthetic valve thrombosis must be considered when the patient is dependent on VA-ECMO support. Early transesophageal echocardiographic examination of the bioprosthetic valve may be helpful and contribute to surgical decision-making.

## Background

Bioprosthetic valve thrombosis is not an uncommon complication and can occur several years after surgery [1]. Acute thrombosis immediately after bioprosthetic valve replacement can also occur, and earlier detection and intervention affect patient outcomes [2, 3]. We present a case of mitral stenosis due to bioprosthetic mitral valve thrombus during left ventricular assist device (LVAD) implantation.

## Case presentation

**Fig. 1:**
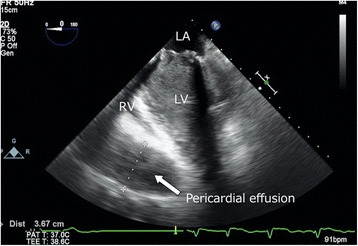
Mid-esophageal four-chamber view showing compression of the right ventricle due to pericardial hematoma. *LA* left atrium, *LV* left ventricle, *RV* right ventricle

**Fig. 2:**
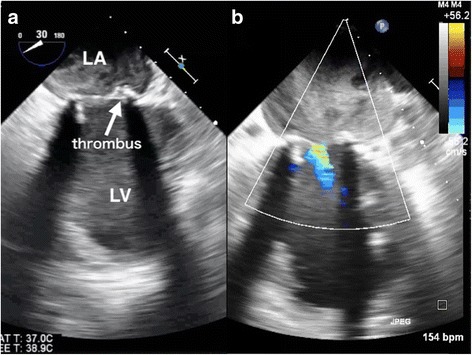
**a** Immobilization of the bioprosthetic mitral valve due to thrombus was observed. Left atrial spontaneous echo contrast was also seen. **b** The thrombus was obstructing transmitral inflow, as seen in the aliased flow pattern using color flow Doppler. *LA* left atrium, *LV* left ventricle

**Fig. 3:**
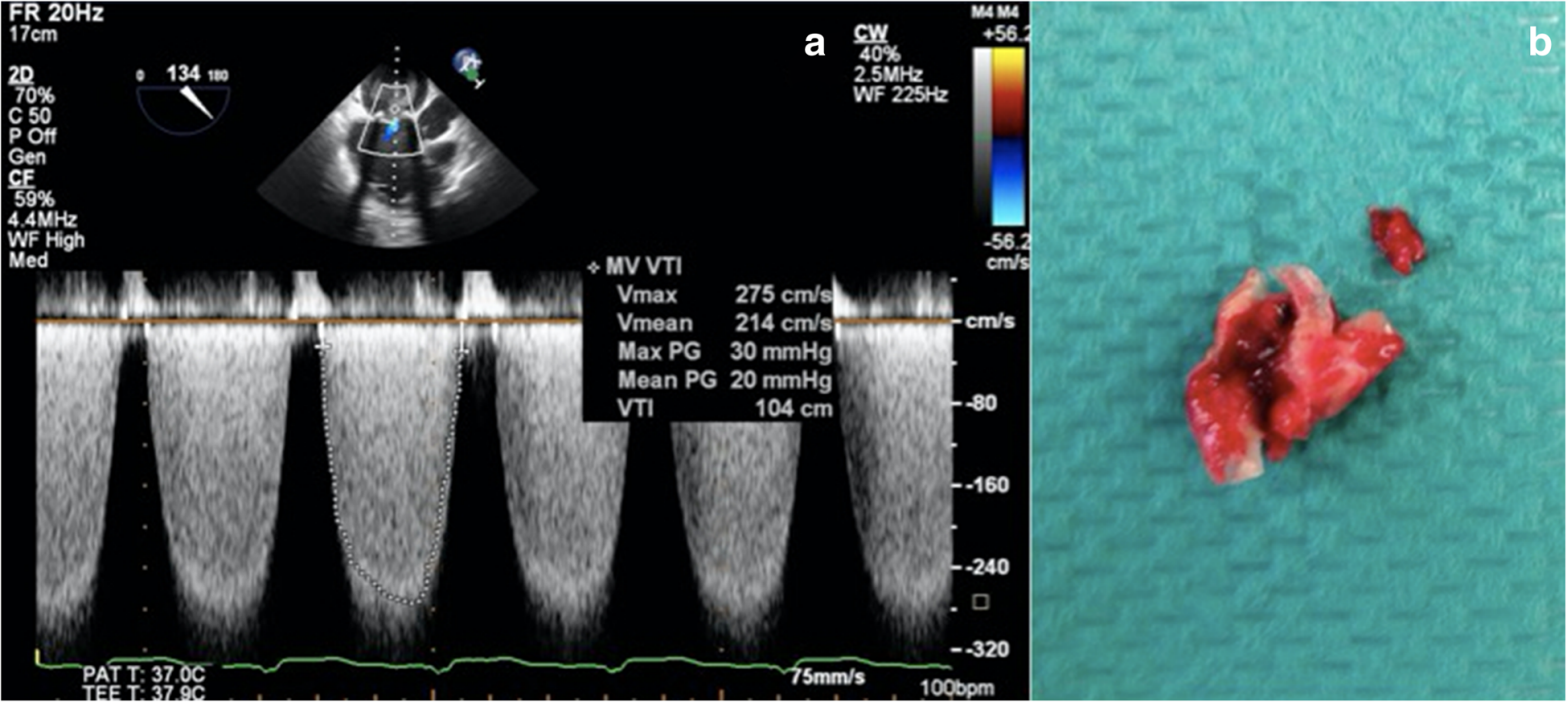
**a** Mid-esophageal long-axis view with continuous-flow Doppler showing a mean mitral pressure gradient of 20 mmHg, which suggests severe mitral stenosis. **b** Organized thrombus adhered to the bioprosthetic mitral valve

Written informed consent was obtained from the patient for the publication of this report. A 61-year-old man (height, 160 cm; weight, 64 kg) with symptomatic functional mitral regurgitation underwent on-pump beating-heart mitral valve replacement (MVR) with a 27-mm Epic stented tissue valve (St. Jude Medical, St. Paul, MN, USA). He had a history of dilated-phase hypertrophic cardiomyopathy, with a decreased left ventricular ejection fraction of 16% and a severely dilated left atrium (LA) (61 × 61 mm, left atrial volume index of 72 mL/m^2^); he was registered as a candidate for transplantation before MVR. He was weaned off cardiopulmonary bypass (CPB) with intra-aortic balloon pumping support and was transferred to the intensive care unit (ICU). In the ICU, heparin therapy was started after achieving hemostasis and controlled to prolong the activated partial thromboplastin time to approximately twice that of the control value over days. On postoperative day (POD) 2, he experienced atrial fibrillation and ventricular tachycardia storm and required venoarterial extracorporeal membrane oxygenation (VA-ECMO) support. He was dependent on VA-ECMO (pump speed of 3300 revolutions/min with a blood flow rate of 4.2 L/min) because of extremely low cardiac output. Transthoracic echocardiography (TTE) examination in the ICU revealed pericardial hematoma, decreased cardiac contractility, and poor opening of the aortic valve, but it could not point out the abnormality of the bioprosthetic mitral valve due to poor echocardiographic images. His clinical status did not improve, and he was scheduled for surgical treatment of pericardial hematoma and DuraHeart (Terumo Heart, Inc., Ann Arbor, Michigan, USA) LVAD implantation as bridge to transplantation to prevent multi-organ dysfunction on POD 9. Transesophageal echocardiography (TEE) before surgical incision revealed right ventricular collapse. Our initial analysis indicated that the hematoma was a part of the cause of the persistent hemodynamic instability (Fig. 1). After removing the hematoma with a median sternotomy, central venous pressure decreased from 17 to 12 mmHg, and the VA-ECMO flow rate decreased incrementally to evaluate cardiac function; pulmonary artery pressure (PAP) drastically increased to 55/35 mmHg. In addition, a large amount of pink blood-stained secretions were expectorated through the endotracheal tube. Additional TEE examination revealed a poor opening of aortic valve, spontaneous echo contrast in the LA, immobilization of the bioprosthetic mitral valve in diastole (Fig. 2a), and abnormal transmitral inflow by color flow Doppler study (Fig. 2b). Continuous-wave Doppler study estimated a mean mitral pressure gradient of 20 mmHg (Fig. 3a). After consultation with surgeons about the possibility of mitral valve thrombus, it was decided that an unscheduled left atriotomy should be performed after CPB initiation, followed by aortic cross-clamp. Direct inspection through the LA revealed an organized thrombus attached to the bioprosthetic valve, which was the cause of valve immobilization (Fig. 3b). After removal of the thrombus, an LVAD was implanted and a temporary right ventricular assist device (RVAD) with a membrane oxygenator was also inserted because of severe pulmonary edema and possible right heart failure after LVAD implantation. CPB was weaned off using dobutamine (5 μg/kg/min), adrenaline (0.05 μg/kg/min), milrinone (0.2 μg/kg/min), and nitric oxide inhalation (20 ppm) support. TEE before weaning off CPB confirmed normal opening of the bioprosthetic mitral valve, and pulsed-wave Doppler examination revealed a mitral pressure gradient of 3 mmHg. The patient was successfully weaned off temporary RVAD 4 days later without an episode of severe right ventricular failure and is waiting for a heart transplant.

## Discussion

The bioprosthetic valve was thought to be superior to the conventional mechanical valve in terms of having low frequency of thrombosis previously [4]. However, recent date suggest that bioprosthetic valve thrombosis is a more common complication than previously reported and the overall occurrence of thrombosis is 11.6% [1]. Also, acute thrombosis after bioprosthetic valve replacement is a possible complication. There have been several case reports of bioprosthetic mitral valve thrombosis causing significant hemodynamic instability [2, 3, 5, 6]. In our case, we initially considered that the hemodynamic compromise might be slightly improved to eliminate pericardial hematoma and low cardiac function. Furthermore, poor TTE images under mechanical ventilation in the ICU masked the presence of thrombus adhering to the mitral valve. The removal of the hematoma and decreasing VA-ECMO flow rate on trial allowed us to inspect the mobility and configuration of the bioprosthetic valve under increased right ventricular output, which further elucidated the cause of mitral stenosis (MS) accompanied by a rapid increase in PAP and pulmonary edema.

To evaluate the bioprosthetic mitral valve, the following TEE examinations are recommended: confirm characterization of the valve morphology and leaflet movement by both two- and three-dimensional imagings and find transmitral inflow and regurgitation patterns by color flow Doppler, as well as pressure gradients (mean and peak) and estimates of mitral valve areas using either continuous or pulsed-wave Doppler.

Risk factors for prosthetic mitral valve thrombosis have been reported as atrial fibrillation, left atrial dilatation, low cardiac output syndrome, hypercoagulability such as heparin-induced thrombocytopenia, and lack of anticoagulation [2, 3, 5, 6]. In our patient, several risk factors for thrombosis were seen, such as a dilated left atrium, low cardiac function, and postoperative paroxysmal atrial fibrillation, in the ICU. We considered that the poor opening of both mitral and aortic valves due to low flow secondary to cardiogenic shock and retrograde perfusion by VA-ECMO synergistically induced bioprosthetic mitral valve thrombosis and valve failure while using proper anticoagulation therapy. VA-ECMO decreases left ventricular preload and increases afterload, leading to blood stasis in the left heart, and it can put the patient at potential risk for bioprosthetic valve thrombosis [2, 5]. The restoration of blood flow from the right ventricle to the left heart through the pulmonary artery after pericardial hematoma removal and decreasing ECMO pump flow provided clinical evidence of MS before LVAD implantation.

## Conclusions

The possibility of bioprosthetic valve thrombosis must be considered when the patient is completely dependent on VA-ECMO support. A closed aortic valve is a sign of blood stagnation in the left heart and of the possibility of thrombosis. Early TEE monitoring of the bioprosthetic valve would be helpful for intraoperative surgical decision-making and contribute to better patient outcomes.

## Abbreviations

CPB: Cardiopulmonary bypass
ICU: Intensive care unit
LA: Left atrium
LVAD: Left ventricular assist device
MS: Mitral stenosis
MVR: Mitral valve replacement
PAP: Pulmonary artery pressure
POD: Postoperative day
RVAD: Right ventricular assist device
TEE: Transesophageal echocardiography
TTE: Transthoracic echocardiography
VA-ECMO: Venoarterial extracorporeal membrane oxygenation
